# Oligonucleotide-Based Therapies for Renal Diseases

**DOI:** 10.3390/biomedicines9030303

**Published:** 2021-03-16

**Authors:** Fernando Cartón-García, Cassondra Jeanette Saande, Daniel Meraviglia-Crivelli, Rafael Aldabe, Fernando Pastor

**Affiliations:** 1 Molecular Therapeutics Program, Center for Applied Medical Research, CIMA, University of Navarra, 31008 Pamplona, Spain; fcartongarc@unav.es (F.C.-G.); dmeraviglia@alumni.unav.es (D.M.-C.); 2 Gene Therapy and Regulation of Gene Expression Program, Center for Applied Medical Research, CIMA, University of Navarra, 31008 Pamplona, Spain; csaande@unav.es; 3 Instituto de Investigación Sanitaria de Navarra (IDISNA), Recinto de Complejo Hospitalario de Navarra, 31008 Pamplona, Spain

**Keywords:** chronic kidney disease, kidney, oligonucleotide therapeutics, kidney delivery, nanocarrier, nanoparticle, aptamer

## Abstract

The global burden of chronic kidney disease (CKD) is increasing every year and represents a great cost for public healthcare systems, as the majority of these diseases are progressive. Therefore, there is an urgent need to develop new therapies. Oligonucleotide-based drugs are emerging as novel and promising alternatives to traditional drugs. Their expansion corresponds with new knowledge regarding the molecular basis underlying CKD, and they are already showing encouraging preclinical results, with two candidates being evaluated in clinical trials. However, despite recent technological advances, efficient kidney delivery remains challenging, and the presence of off-targets and side-effects precludes development and translation to the clinic. In this review, we provide an overview of the various oligotherapeutic strategies used preclinically, emphasizing the most recent findings in the field, together with the different strategies employed to achieve proper kidney delivery. The use of different nanotechnological platforms, including nanocarriers, nanoparticles, viral vectors or aptamers, and their potential for the development of more specific and effective treatments is also outlined.

## 1. Introduction

Recent studies have estimated that chronic kidney diseases (CKDs) affect around 850 million people worldwide (one in ten adults). The global burden of CKD is increasing and is projected to become the fifth most common cause of years of life lost globally by 2040 [1]. Since CKD is mostly irreversible and progressive, patients who advance to end-stage renal disease (ESRD) require dialysis or renal transplantation, which negatively affect quality of life and have a large impact on healthcare systems. It has been estimated that the costs of dialysis and transplantation consume 2–4% of annual healthcare budgets in high-income countries [1,2]. Moreover, kidney transplantation is associated with a high risk of morbidity and mortality, after rejection, infections, and cancer development, as a consequence of the continuous immunosuppression required [3]. Therefore, kidney disease has a major effect on global health and deserves greater attention for the development and improvement of new detection methods and treatments.

Oligonucleotide (ON) therapeutics, such as those based on antisense oligonucleotides (ASOs), small interfering RNA (siRNA), microRNA (miRNA), aptamers, and decoys, are promising agents that have gained importance during the last decades. As of January 2020, ten oligonucleotide drugs have received regulatory approval from the United States Food and Drug Administration (FDA) and dozens are under clinical trials [4,5]. However, a major obstacle that still hampers the development of new oligonucleotide-based therapies is the difficulty in directing them to specific organs. The kidneys are highly vascularized organs that receive up to 25% of cardiac output, and are susceptible to targeting by most systemic administration routes. Additionally, the glomerular filtration barrier has evolved to filter molecules smaller than 50 kDa in size, which includes the majority of oligonucleotides commonly used in therapeutics, allowing their access to the tubular epithelium. However, this route mostly favors targeting of the liver and other peripheral organs, such as the spleen, due to its vascularized anatomy and scavenging functions. Indeed, at least half of the approved oligonucleotide-based drugs have been developed for liver therapy [4,5]. The unresolved problem of non-specific and off-target effects is a second major obstacle yet to be overcome by improving delivery methods. Importantly, toxicity, and side-effects of oligonucleotides have already been described, including inhibition of unspecific genes, oversaturation of the endogenous small RNA processing pathways, or non-complementary binding of the oligonucleotide to unintended RNAs with a sequence similar to the target RNA [6,7,8,9,10]. In this review, we will outline the different oligotherapeutic strategies developed to date for the treatment of renal diseases, with a specific focus on the delivery methods and nanotechnological platforms developed, and their potential to achieve efficient kidney delivery.

## 2. Oligonucleotides Used in Therapeutics

Oligonucleotide therapies have received considerable attention in recent years, mainly because of their advantages over conventional treatments. Contrary to traditional small molecule drugs, which typically combat disease pathology by modulating the downstream pathways of a disease-causing gene, oligonucleotide-based therapies may directly modify the gene encoding intermediates at fault by targeting DNA or mRNA precursors. Therefore, ONs can also be effective against a wide variety of targets, including proteins and posttranslational protein modifications, carbohydrates, lipids, and metabolites, by directly targeting them with aptamers, or by modulating gene expression. Their versatility is derived from their simple structure, easy synthesis, and the possibility of a rational design. In contrast to conventional drugs that are usually limited to binding specific protein pockets or active sites, DNA and RNA targeting is mostly based on sequence recognition or the presence of unique three-dimensional conformations that allow ONs to potentially bind any target molecule [11,12]. To date, many different oligonucleotide-based therapeutic strategies have been designed, including both DNA and RNA ONs. However, the active roles that RNAs play in cell biology and metabolism, together with our increasing understanding of their role in gene expression and endogenous regulatory machinery, make RNA-based therapeutics the preferred option for their use in medicine [13].

One of the major drawbacks of using extra-cellular oligonucleotides, especially those based on RNA, is their susceptibility to degradation by nucleases and poor pharmacokinetics. To overcome these limitations, such drugs often include chemical modifications of their backbone and nucleobases that increase stability, enzymatic resistance, and efficacy, which will be explained in further detail in the next chapter of this review. A notable advantage of targeting the kidney is that oligonucleotide therapies are rapidly cleared from the circulation via renal filtration, favoring their biodistribution in the kidney over other organs [14]. Here, we will outline the main strategies employed in the development of oligonucleotide-based therapies and the most recent advances for their use in renal diseases, which are also summarized in Table 1.

### 2.1. RNA-Based Strategies

#### 2.1.1. siRNA

Short interference RNAs are short (20–27 nucleotides) double-stranded RNAs that target and degrade mRNA in a sequence-specific manner. The guide (antisense strand) is loaded onto Argonaute 2 protein (AGO2), forming the RNA induced silencing complex (RISC), whereas the sense strand is cleaved. The guide strand targets the specific mRNA by complete complementarity and AGO2 catalyzed mRNA cleavage. The RISC and guide strand can be recycled to target multiple mRNA molecules leading to efficient gene silencing [11,13,59]. Alternatively, siRNAs can also be designed to target long non-coding RNAs (lncRNAs), often involved in transcriptional repression, reversing the effects of this negative regulation [4]. siRNAs can sometimes be encoded in the form of short-hairpin RNAs (shRNAs), which are usually delivered to the cell by transduction with viral vectors and, in some cases, integrated into the host genome. shRNA is first expressed as a miRNA and processed into a siRNA duplex by the enzymes Drosha and Dicer, which then follows the same interference mechanism previously described [13]. Two siRNA-based therapies, Patisiran and Givosiran, have already been approved by the regulatory agencies in May 2020, both targeting the liver [4,5]. Interestingly, siRNA technology has also been explored for kidney diseases, showing potential as a therapeutic agent as well as contributing to the understanding of the molecular mechanisms of renal diseases (Table 1) [60]. One of the earliest studies successfully demonstrated the feasibility of using a siRNA-based therapy to ameliorate glomerular sclerosis in a mouse model of glomerulonephritis, by modulating the transforming growth factor beta (TGFβ) pathway as result of *Mapk1* silencing [17]. Likewise, Morishita et al. [19] prevented renal fibrosis by using a siRNA against *Smad4,* suggesting it could be a crucial therapeutic target for renal fibrosis in vivo. Other studies using siRNA-based drugs have focused on reducing the extent of acute kidney injury (AKI), an unavoidable side effect of numerous medical treatments and surgical procedures which deprive the kidney of oxygen. For instance, Glebova et al. [61] evaluated the potential of 53 different siRNA targets, mainly related to apoptosis, inflammation and immune rejection pathways after ischemia-reperfusion caused by transplantation. This approach is still under development but has already shown promising results in a mouse model [22]. In a similar study, the authors effectively evaluated the prophylactic role of siRNA targeting meprin-1β and p53 expression in a cisplatin-induced murine model of AKI. These two proteins play key roles in depolarization and apoptosis after kidney injury [23]. Narváez et al. [25] also demonstrated that the administration of a siRNA therapy against *Cd40* in a mouse model of AKI induced by unilateral ureteral obstruction (UUO) significantly reduced inflammation and promoted kidney repair. Importantly, upon demonstration of a lack of reduction in megalin protein expression in vivo using a siRNA, another recent study has pointed out that the use of siRNA-based therapies in kidney diseases might, indeed, be more suited to prevention of upregulation than reduction of constitutive baseline mRNA expression [24]. Although there is still scope for improvement, siRNA-based therapies in the kidney have shown promising results, especially in the prevention of AKI where some clinical trials have already been carried out [60].

#### 2.1.2. saRNA

Small activating RNAs are double-stranded RNAs of 21 nucleotides in length that possess two nucleotide overhangs on both ends. Comparable to siRNA, saRNAs are loaded onto AGO2, where the sense strand in cleaved. Then, the saRNA–AGO2 complex is translocated to the nucleus, binding to complementary promoter regions and recruiting key elements for transcription initiation [62]. Thus, saRNA has an identical structure and chemical components as siRNA, but its biological function is the opposite of siRNA, since it acts to enhance gene transcription. The use of saRNA with therapeutic purposes has been recently tested in an in vivo model of ethylene glycol (EG)-induced calcium oxalate (CaOx) kidney crystal formation in rats. Using this approach, the authors significantly enhanced the expression of *Trpv5*, a key protein mediating calcium transport and reabsorption in the kidney, and achieved a reduction in CaOx crystal formation by promoting calcium reabsorption [34].

#### 2.1.3. miRNA

miRNA constitute a class of single-stranded non-coding RNAs (ncRNAs) with a length of approximately 22 nucleotides after maturation. Their natural biogenesis starts in the nucleus, where they are transcribed as pri-miRNAs and cleaved into pre-miRNAs (~70 nucleotides in length) by a multiprotein complex mainly comprised of the Drosha and Dcrg8 proteins. Double-stranded pre-miRNAs are then exported to the cytoplasm and processed by Dicer into mature miRNAs (~22 nucleotides in length) [63]. For their mechanism of action, miRNAs are loaded onto AGO2 to form RISC, guiding the complex to its complementary binding site in the target transcript, commonly found in the 3′ untranslated region (UTR). In contrast with siRNAs, miRNAs typically bind with partial complementarity and usually promote translational repression by triggering mRNA decay through deadenylation and decapping. Different miRNAs can bind to the same transcript by overlapping or non-overlapping sites [11,13]. In general, there are three approaches to developing miRNA-based therapeutics: (1) anti-miRNA oligonucleotides (AMOs) or miRNA antagomirs, also known as antisense oligonucleotides (ASOs), which antagonize endogenous miRNAs by steric blocking of the miRNA within the RISC complex [4,11]; (2) miRNA sponges, which are genetically engineered competitive miRNA inhibitors designed by insertion in tandem of multiple binding sites of targets of analogous miRNAs or mRNAs [64]; (3) miRNA mimics, which are engineered double-stranded miRNAs that replace, improve or supplement the function of native miRNAs [11,59]; (4) target site blockers (TSB), designed to recognize and mask the regulatory sequences of miRNAs within a specific mRNA, with the potential to maintain the rest of the mRNA network unaffected [4].

miRNAs play an important role in the negative regulation of post-transcriptional gene expression. Consequently, aberrant miRNA expression is implicated in the development and progression of numerous diseases, and multiple families of miRNAs are shown to be dysregulated in kidney disease. The role of miRNAs in molecular pathology has already been reported in AKI and kidney transplantation [65,66], polycystic kidney disease (PKD) [67,68], and renal fibrosis [69,70], which represents the final outcome and most relevant pathological event of CKD. This, together with the fact that miRNAs can be detected in exosomes in plasma and urine, indicate them as suitable and attractive new biomarkers for diagnostic purposes and disease monitoring [71,72]. Importantly, up/downregulated miRNAs also represent novel therapeutic targets for kidney diseases, whose potential has already begun to be explored in animal models (Table 1). One of the earlier studies was carried out by Chau et al. [35], who successfully used an anti-miR21 antagomir, which limited injury and kidney fibrosis in two murine models of AKI. Similarly, renal fibrosis was also ameliorated by an anti-miR192 antagomir in a mouse model of diabetic nephropathy (DN) [36]. The use of miR204 and miR211 mimics has also proved effective at reducing the severity of kidney injury in a mouse model of systemic candidiasis [37] and inhibition of miR107 using an antagomir significantly prevented tubular cell injury in a mouse model of AKI induced by sepsis [39]. Moreover, the use of miRNA-based approaches has also shown potential in the treatment and understanding of the basis of ischemic-kidney injury, as demonstrated by a collection of recent studies (Table 1). One of the most common causes of ischemic AKI is transplantation. In that regard, a recent study has demonstrated the applicability of using antisense technology against the miR182-5p target to improve kidney function and morphology, employing a model of ischemic AKI in rats [40]. In addition, Wei et al. [41,42] elucidated the molecular mechanisms of miR489 and miR668 in the protection of the kidney during ischemia, indicating the possibility of using these miRNA or miRNA mimetics as therapeutic agents. Similarly, another study demonstrated the protective role of miR199a-3p in an ischemia-reperfusion model in vivo [45]. Taken together, these findings are promising for future evaluation of the clinical utility of miRNA mimetics and inhibitors targeting key pathologic renal pathways. However, translation of preclinical findings is sometimes complicated, as a deep understanding of the miRNA regulatory networks underlying the disease is needed. In some cases, targeting key points in the same network may prove more effective. Additionally, most miRNAs are regulated in a cell-type or organ-specific manner; thus, the possibility of off-target and undesired effects in unrelated organs is high. This problem could explain why few investigations using miRNAs-based therapies move forward to the clinical stage. In fact, only four miRNA-based therapies have reached clinical development, two of them dedicated to renal disease [66]. One of the drugs was developed for Alport nephropathy, a genetic disorder characterized by chronic glomerulonephritis that progresses to end-stage renal disease in young adult life. The antagomir against miR21 was effectively evaluated in a mouse model, where the animals displayed substantially milder disease and significantly improved survival after treatment [38]. The drug RG012 targeting miR21 is currently undergoing a phase 2 clinical trial (clinical trial identifier NCT02855268). The second drug is RGLS4326, an antagomir inhibiting miR17 developed for the treatment of autosomal dominant PKD (ADPKD), a genetic disorder caused by mutations in either *PKD1* or *PKD2* genes resulting in hyperproliferation of the renal tubular cells and cyst formation [43]. Treatment with RGLS4326 attenuated cyst growth in several PKD mouse models and human ADPKD models in vitro and is now in a phase 1 clinical trial (clinical trial identifier NCT04536688).

### 2.2. RNA/Protein-Based Strategies (CRISPR)

The development of the revolutionary CRISPR/Cas9 gene editing technology is driving the progress of RNA therapeutics forward in a similar way. This system, initially discovered as a form of acquired immunity in bacteria and archaea, consists of a protein (CRISPR-associated nuclease Cas9) and an oligonucleotide guide RNA (sgRNA). While the gRNA directs the nuclease to a specific genomic location adjacent to a protospacer adjacent motif (PAM) sequence, correct base-pairing activates Cas9 nuclease domains, which, cut the DNA, resulting in a double strand-break. In an attempt to repair the damage, eukaryotic cells can use two different mechanisms, nonhomologous end joining (NHEJ) and homology-directed repair (HDR). The NHEJ repair mechanism is prone to introduce small insertion or deletion errors, causing frameshift mutations and leading to gene knockout by disruption of the open reading frame. Conversely, when a donor template is introduced, it can be utilized by the HDR mechanism and harnessed to introduce a new sequence bearing a mutational correction or sequence knock-in in the desired loci [73,74]. A great advantage of the system, as consequence of its modularity, is that it allows for the testing of many different potential sgRNA while maintaining the protein component invariant. Importantly, although this system was initially developed to target DNA, RNA-targeting and interference has also been possible due to the development of new engineered nucleases such as Cas13 and RNA-targeting Cas9 (rCas9). Similarly, nuclease deficient Cas9 variants (dCas9) restricted from generating DNA breaks have been fused to transcriptional activation (VPR) [75], silencing proteins (KRAB) [76], or epigenetic modifiers [73,74], which can then be targeted to specific gene promoters, regulating gene expression. Catalytically inactive Cas9 and Cas13 variants have also been fused to other types of functional domains, such as base-pair editors or deaminases able to catalyze A-T to G-C transitions, allowing for single-base edition at the DNA and RNA level without the need to generate double or single-strand breaks [74], or reporter proteins, to visualize DNA or RNA [73]. Nonetheless, despite the fact that the CRISPR/Cas9 system has emerged as a promising platform with a wide variety of applications in biology research and therapeutics of human disease, efficient and safe delivery of its components to target cells in vivo remains challenging [77].

The use of the CRISPR/Cas9 system as a therapeutic approach for renal diseases has great potential, as a significant proportion of these diseases, such as autosomal dominant PKD or Alport syndrome, arise as consequence of genetic mutations. Nevertheless, gene editing in solid organs still faces the challenge of effective delivery to specific cells or tissues. Thus, the use of this tool in kidney research has so far been limited to its application in the development of novel in vitro (using human organoids) and in vivo models of renal disease. Such models are very useful in understanding the molecular mechanisms underlying renal diseases, as well as in the identification of new genes responsible for their progression and that could represent potential therapeutic targets [78,79]. Another exciting potential use of CRISPR/Cas9 technology is focused on expanding the available sources of kidneys for transplantation. Some authors have proposed the possibility of transplanting organs from other species such as pigs (xenotransplantation), an approach that, to this day, would lead to an extreme human immune response and rejection of the donor organ. However, CRISPR/Cas9 has appeared as a promising tool that could circumvent this limitation. In this direction, some authors have already employed CRISPR/Cas9 to genetically modify swine eggs in order to generate animals lacking carbohydrate xenoantigen, whose recognition by human and non-human primate antibodies was effectively diminished [80], or to generate I MHC null pigs [81]. The first demonstration of the feasibility of this approach was published by Higginbotham et al. [82], where the authors achieved effective pig-to-primate long-term transplantation (>125 days).

### 2.3. DNA/RNA-Based Strategies

#### 2.3.1. Antisense Oligonucleotides (ASOs)

Antisense oligonucleotides are synthetic, small, single-stranded nucleic acids (18–30 nucleotides) designed to target mRNA sequences and inhibit gene expression. As previously described, ASOs can also target lncRNAs that negatively regulate transcription, leading to “unsilencing” or the activation of gene expression [4]. In general, ASOs act in two different ways: (1) forming RNA:DNA heteroduplexes with complementary mRNA, in which the RNA strand is recognized and cleaved by RNase H; (2) steric blocks of splicing and translation, which bind to a precursor mRNA (pre-mRNA), but do not form RNase H substrates. Regarding the formation of RNA:DNA heteroduplexes, RNase H is active in both the cytoplasm and the nucleus, enabling targeting of nuclear transcripts. Many of the RNase ASOs are designed including bases of different chemistries, as for example gapmers, whereby a central DNA-based “gap” is surrounded by RNA-based “wings”, which constitute chemically modified flanking regions that promote target binding [11,83]. In the case of steric blocking, ASOs bind to specific splicing signals that are important for RNA-RNA and/or RNA-protein interactions and spliceosome formation triggering exon exclusion or retention. In order to prevent the potential formation of RNA:DNA heteroduplexes, steric blocking ASOs are generally designed to contain chemically altered riboses or as phosphorodiamidate morpholino oligomers, carrying a modified heterocyclic backbone ring. Consequently, one of its most common applications includes the restoration of a translation reading frame or, on the contrary, the disruption of translation of a target gene by modulating splicing decisions [11,83]. To date, six different drugs based on ASO technology have been approved by the regulatory agencies and extensive research regarding new sequences and strategies is published every year, including approaches targeting renal disease (Table 1). Accordingly, in 2007, Guha et al. [49] published one of the first studies using an ASO-gapmer targeting connective tissue growth factor (*Ctgf*), an upregulated mediator of the TGFβ pathway that contributes to the pathology of DN. The authors demonstrated that inhibition of *Ctgf* expression attenuated DN in a mouse model. Another study using an ASO-gapmer to silence *Kras* in a rat model of renal fibrosis caused by UUO demonstrated marked amelioration of the pathologic phenotype [50]. Ravichandran et al. [51,52] have also shown promising preclinical data in the treatment of ADPKD using ASOs directed at inhibiting the target of rapamycin complex (*mTORC*). mTORC inhibitors have already been explored for the treatment of PKD in rat models and in human subjects, but have showed limited effectiveness in preventing disease progression [51]. Hence, to improve disease outcome, in one of their studies the authors used a novel ASO-gapmer that successfully achieved inhibition of both *mTORC1* and *mTORC2* in mice littermates, resulting in a significant reduction in tubular cell proliferation and cyst growth, as well as improved kidney function [51]. Similarly, in a second study, the authors also demonstrated the effectiveness of another ASO-gapmer targeting the renin-angiotensin system, upregulated in PKD. By inhibiting angiotensinogen, the authors observed a significant decrease in proinflammatory cytokines, interstitial fibrosis and cyst volume density in two PKD mouse models [52]. ASOs have also been investigated in a limited number of studies for their potential in targeting the regulation of renal tumor development and metastasis in both in vitro and in vivo models of renal cell carcinoma (RCC) [46,48,84,85]. For example, Shi and Siemann [46] evaluated the anti-tumor efficacy of an ASO directed against vascular endothelial growth factor (VEGF), a key factor implicated in tumor angiogenesis. The authors demonstrated reduced VEGF expression and impaired cell proliferation and migration in a renal cell carcinoma cell line (Caki-1), and slower tumor growth in mice bearing RCC xenografts, following treatment with the ASO directed against VEGF. In summary, these studies indicate that the use of new therapeutic tools based on ASOs are feasible for the treatment of renal diseases.

#### 2.3.2. Aptamers

Aptamers are short single stranded DNA or RNA molecules (of 20–100 nucleotides in length) that fold into defined three-dimensional structures, which enables them to bind targets with high affinity and specificity, similar to that attributed to antibodies (in the low nanomolar or picomolar range). In contrast to other nucleic acids, aptamers usually are not rationally designed but are generated using an iterative methodology known as systematic evolution of ligands by exponential enrichment (SELEX). Using this approach and after several rounds of enrichment, aptamers can be selected for a wide variety of targets, including a single amino acid mutation or conformational isomers [86,87,88]. One of their main advantages compared to other nucleic acid therapies is that aptamers can exert their activity extracellularly by targeting receptors or ligands on the cell surface. Nonetheless, aptamers that target intracellular molecules, such as transcription factors, exist as well [89]. Hence, many aptamers have been selected to exhibit a therapeutic effect by themselves through antagonism or agonism of specific ligands/receptors. This is the case of the drug pegaptanib, the first aptamer-based drug approved by the FDA, which binds and counteracts the action of VEGF in the treatment of age-related macular regeneration [90,91,92]. Importantly, as consequence of their small size and high tissue penetration, aptamers can also be used as delivery cargoes following conjugation with other drugs, such as proteins, small molecules or other oligonucleotides [4,86,93,94,95]. This option, together with high target specificity, represents a huge advantage in the use of oligonucleotide-based therapies, for which delivery problems and off-target effects are still among its main drawbacks precluding further development. Indeed, their applicability to specifically target renal cells has already been probed in a recent study performed by Ranches et al. [96]. The authors employed renal proximal tubule cells (RPTEC/TERT1) stimulated with a mix of pro-fibrotic and pro-inflammatory cytokines in order to reproduce tubulointerstitial fibrosis and inflammatory conditions occurring in CKD. Then, by using a cell-SELEX approach, only cell-internalized DNA aptamers that bound cells with a pathophysiologically altered phenotype were selected. The aptamers obtained displayed a significantly higher binding specificity for those cells compared to non-stimulated RPTEC cells. This study demonstrates, for the first time, which aptamers could represent a valuable tool for the development of new diagnostic tools and targeted therapies to treat renal diseases. Furthermore, as previously mentioned, aptamers can also be selected by their ability to activate/inhibit specific receptors and modulate intracellular pathways. This possibility has been also investigated for the treatment of renal injury in a number of recent studies (Table 1). Advanced glycation end products (AGEs) are irreversibly cross-linked adducts resulting from non-enzymatic reactions of reducing sugars with amino groups of proteins and lipids. Interaction of AGEs with their receptor, RAGE, generates oxidative stress, inflammation and fibrosis, altering tissue architecture and leading to renal injury. The development of DNA aptamers against RAGE effectively suppressed AGE-induced oxidative stress and ameliorated renal damage in a streptozotocin-induced rat model of DN [55]. Subsequently, the same authors also demonstrated the effectiveness of the RAGE aptamer in the prevention of hypertensive renal injury in a mouse model [57]. Additionally, another study developed a DNA aptamer binding periostin, an extracellular matrix protein upregulated in DN and renal fibrosis. Using a mouse model of DN, aptamer treatment significantly attenuated the pathologic phenotype [56]. Aptamers have also been developed with high binding affinity and specificity to RCC cell lines [58,97], with the potential for their use in the identification and targeting of RCC. Zhang et al. [58] identified an aptamer (SW-4) with selective binding to the RCC 786-O cell line that inhibited cell proliferation by cell cycle arrest. Furthermore, the authors demonstrated that SW-4 retained targeting specificity to RCC in vivo following tail vein injection of the aptamer in a 786-O xenograft mouse model, and also maintained recognition of clinical RCC tissue samples. Notably, the well-known anticancer aptamer AS1411 entered a phase 2 clinical trial in patients with metastatic RCC (clinical trial identifier NCT00740441), although the authors reported low overall efficacy [98]. In summary, aptamers represent promising tools for their application in renal diseases as therapeutic agents or targeting molecules; however, limited studies have been reported thus far. Moreover, the aptamers previously described were selected by in vitro-SELEX, and may show some limitations when translated to complex in vivo organisms. Alternatively, the use of in vivo-SELEX approaches, employing whole organisms, has become increasingly relevant in recent years [86]. The use of animal models of renal diseases for in vivo-SELEX purposes could open new doors for the development of aptamers with enhanced therapeutic or targeting potential.

### 2.4. DNA-Based Strategies

#### Transcription Factor Decoy (TFD)

TFDs are short double-stranded DNA molecules that are designed to mimic the binding site of a target transcription factor. Thus, they specifically bind and sequester relevant transcription factors interfering with the expression of the genes that they regulate [11,99]. The potential of TFD in renal disease has been successfully demonstrated by Chae et al. [53] in an in vivo rat model of UUO-induced renal fibrosis (Table 1). The authors used a TFD designed to sequester the Sp1 transcription factor that regulates the expression of TGFβ1, whose role in tubulointerstitial fibrosis is well described [53]. After treatment, Sp1-TFD significantly attenuated extracellular matrix expression genes and interstitial fibrosis during the progression of obstructive nephropathy. Likewise, another study also used E2F and NFκB TFD to ameliorate glomerular injury and inflammation in a rat model of glomerulonephritis, showing positive results [54]. Nevertheless, in spite of the fact that TFDs have demonstrated good results in preclinical models of renal diseases, only those designated for cancer therapy are close to being translated into clinical development [100].

## 3. Overcoming Delivery Problems

Despite the increasing number of oligonucleotide-based therapeutic strategies published every year, single-stranded DNA and RNA oligonucleotides have properties that complicate drug development. Some of the major problems are: (1) degradation by nucleases when they are introduced into biological systems; (2) the presence of non-specific and off-target effects; (3) innate immune activation; (4) unfavorable biodistribution and pharmacokinetic properties; (5) delivery problems to target tissues/cells and poor uptake through cell membranes; (6) sub-optimal binding affinity for complementary sequences. Consequently, the great progress achieved in the field over the recent years has been concurrent with the development of new nucleic acid analogs with improved metabolic stability, reduced toxicity and immunogenicity, as well as new strategies aiming to increase target delivery and specificity [4,11,59]. In this part of the review, we will discuss the latest advances with a focus in the limitations of oligotherapeutics specifically targeted to the kidney.

### 3.1. Chemical Modifications to Improve Stability and Biodistribution

Oligonucleotide drugs have the potential to induce innate immune responses, triggering the production of type I interferons, proinflammatory cytokines and transient complement cascade activation. Several Toll-like receptors in the cytoplasm are able to recognize double-stranded RNA motifs (TLR3), single-stranded RNA (TLR7 and 8) or unmethylated CpGs (TLR9) [99,101]. Another caveat of oligonucleotides, especially RNA, is an abundance of endo- and exonucleases present in serum and in the cells, which efficiently cleave the phosphodiester bonds and reduce the half-life of these therapeutic agents. Moreover, their size and negative charge impede their diffusion through the plasma membrane [4,102]. Thus, to overcome these limitations, it is necessary to introduce chemical modifications and use accurate “tailored” designs for each case. ONs can be modified by changing the phosphodiester bond by replacing the non-bridging phosphodiester oxygen by sulfur or borane, forming phosphorothioate (PS) or boranophosphate linkages, respectively [103,104]. This modification increases ON stability against nucleases while maintaining its compatibility with RNase H mediated cleavage of RNA. In addition, PS backbones bind to serum proteins, such as albumin and heparin-binding, which serve as carrier scaffolds to increase the circulation time of PS oligonucleotides, slowing clearance by the liver and kidney. Moreover, these modifications increase ON half-lives from minutes to days, augmenting the time available for tissue and cell uptake [14,83]. PS modifications also increase interaction with intracellular proteins, which could also favor their accumulation in certain cellular compartments [105].

Strategies to modify nucleobases and ribose sugars have also been investigated, and some of them have already been applied in the development of oligotherapeutics for renal diseases [38,43]. These modifications aim to enhance stability and target binding affinity while maintaining base pairing and unaltered conformation of the double helix. The use of inverted thymidine residues at the 3′ RNA end has been reported to increase protection against exonucleases, and pyrimidine methylation (5-methylcytidine and 5-methyluridine/ribothymidine) improves binding specificity by increasing the melting temperature of ONs [11,59,90]. Ribose modifications are generally utilized through replacement of the 2′-hydroxyl by at least 13 different groups, with 2′-O-methyl, 2′-O-methoxyethyl and 2′-Fluoro among the most common. These modifications provide resistance to nuclease degradation by blocking the nucleophilic 2′-hydroxyl moiety and increase the thermal stability of complementary hybridization [11,106]. Moreover, cytokine induction or other immunogenic effects caused by oligonucleotides can be abrogated by incorporating some of these modifications, such as the 2′-O-methyl group [107,108,109]. Importantly, 2′-ribose modification of ONs is not compatible with RNase H activity and will not be cleaved. As such, this type of modification is typically introduced in the case of steric block approaches, or in the flanking sequences of gapmers [5]. Similarly, only 2′-O-methyl and 2′-Fluoro modifications are tolerated by the reverse transcriptase and by T7 mutant RNA polymerases, which are commonly used during SELEX. Therefore, chemically modified RNA libraries with 2′-fluoropyrymidines nucleotides are the most highly utilized for aptamer production [90]. Notably, unnatural l-ribose molecules have also been employed for the synthesis of l-ribonucleic acid aptamers or Spiegelmers, preventing their recognition by nucleases and thus conferring excellent in vivo biostability. Nevertheless, the selection of potential L-form RNA aptamers requires obtaining the enantiomer of the target of interest for SELEX, thereby limiting its use for some macromolecules [4,90]. Likewise, the 2′-oxygen can also be linked through bridging carbon atoms to the 4′ carbon of the ribose to form a bridged nucleic acid (BNA). The most commonly used BNA is the locked nucleic acid (LNA), where a methylene bridge links the 2′-oxygen with the 4′ carbon position, substantially strengthening hybridization and increasing melting temperature by as much as 5 °C–10 °C. Enhanced affinity binding allows the use of ONs as short as thirteen nucleotides in length for clinical applications [110]. Unfortunately, these modifications do not result in an improvement in delivery, per se.

**Figure 1:**
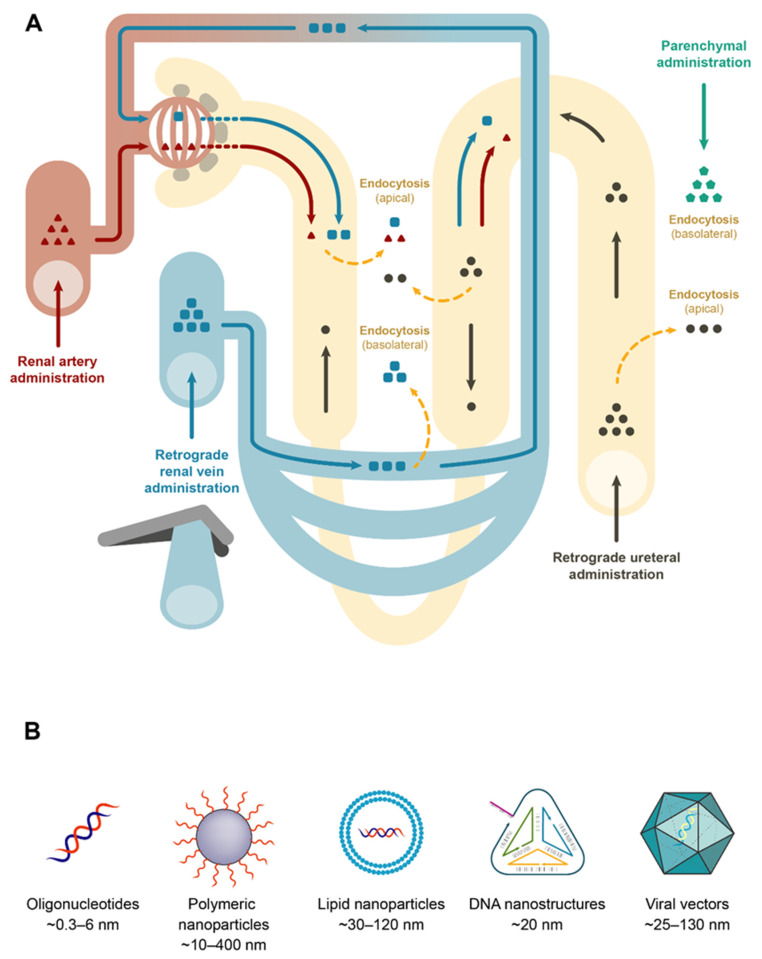
Diagram of the nephron and direct routes of administration. (**A**) Renal artery administration targets the glomeruli, where particles larger than 10 nm are retained by the glomerular pores and basement membrane. Smaller particles that are filtered can be endocytosed in the apical pole of tubular epithelial cells. After retrograde renal vein administration, high hydrodynamic pressure creates transient pores in endothelial membranes, allowing particle leakage and endocytosis by the basolateral pole of tubular epithelial cells. In order to induce a retrograde flow towards the glomerular capillary network, a short period or renal vein clamping is necessary. Small particles that are filtered through the glomerulus can also reach the lumen of the renal tubules. Retrograde ureteral administration targets tubular epithelial cells through the lumen of the renal tubules. Parenchymal administration targets the renal interstitium and the tubular epithelial cells by basolateral endocytosis. (**B**) Types of particles administered to the kidney and their approximate sizes; oligonucleotides (siRNAs, saRNA; miRNAs, ASOs, aptamers); polymeric and lipid-based nanoparticles (lipoplexes, liposomes and exosomes); DNA nanostructures (nanocages, tetrahedron); viral particles (AAV, adenovirus, lentivirus).

The possibility of creating oligonucleotide analogs has represented a crucial step forward in the clinical development of ON therapeutics. Nevertheless, improved stability and tissue retention may give rise to undesirable side effects due to prolonged gene silencing, saturation of the small RNA processing machinery and higher rates of off-target effects. Understanding drug biodistribution and pharmacokinetics is therefore critical in the development of oligonucleotide-based drugs. The majority of ONs are administered through intravenous or subcutaneous injections (Table 1). Following administration, they rapidly distribute in the bloodstream and preferentially accumulate in the liver, spleen and kidneys, mainly due to the vast network of capillaries, endocytic activity and presence of phagocytic cells in these organs. Moreover, as small sized molecules, they are subject to rapid excretion through renal filtration. Consequently, the kidneys benefit from both rapid and vast blood flow and subsequent tubular absorption and constitute a primary site of ON accumulation, accounting for up to 20% of the concentration of the total administered dose [14]. Indeed, some chemically modified ONs, including the clinically evaluated RGLS4326 mir-17 antagomir, have been described to reach tubule cells by both basolateral uptake from the bloodstream and tubular reabsorption [43,60] (Figure 1). ONs are postulated to be reabsorbed from the glomerular ultrafiltrate by receptor mediated-endocytosis through highly expressed receptors such as megalin, which actively participates in the endocytic uptake of a wide variety of molecules [18,111,112] (Figure 1). Of note, even though pharmacokinetics may favor the delivery of ONs to the kidney, a high concentration in this organ can also lead to adverse effects, such as competition with and saturation of receptor-mediated endocytosis in the proximal tubules. As shown by Janssen et al. [113], the use of a 2′ O-methyl phosphorothioate antisense oligonucleotide (Drisapersen) for the treatment of Duchenne muscular dystrophy led to alpha-1-microglobulin proteinuria in pre-clinical and clinical studies, although histopathological evidence from cynomolgus monkeys did not reveal apparent tubular damage. Similarly, several reports have also indicated the potential of modified ONs to induce renal damage despite the low toxicity profile of new generation chemistries and the fact that promising drugs, such as RGLS4326, have been demonstrated to be safe [111,114]. Thus, the potential for renal damage is especially important to bear in mind when designing therapies to target the kidneys.

Apart from using “tailored” designs of the ON chemistries that best suit each purpose, the possibility of creating direct covalent bioconjugates with various moieties that promote target specificity and intracellular uptake is another alternative. Additionally, the implementation of nanotechnology in oligotherapeutics by using nanoparticles (NPs) or other nanocarriers could also protect ONs from degradation and reduce proinflammatory responses.

### 3.2. Improving Kidney Delivery

The use of naked ONs by systemic administration has demonstrated the feasibility of targeting the kidney by this approach and has been the option chosen in many studies, primarily through the use of chemically modified gapmer ASOs and antagomirs (Table 1). Importantly, single stranded ONs (ssONs) display a much more flexible conformation than duplex ONs, where the bases face the interior. This conformation makes them more prone to binding proteins, which increases the probability of cell uptake [112,115]. Consequently, the use of these strategies for kidney targeting requires a thorough evaluation of the potential off-targets and side effects before reaching the clinical arena. It is important to note that the presence of fenestrated endothelium in the kidneys and the possibility to use more direct routes of administration represents a major advantage for the use of large bioconjugates and nanocarriers, reducing retention by other organs (Figure 1).

#### 3.2.1. In Vivo Local Delivery

Based on the anatomical structure of the kidneys, local delivery to the different compartments can be achieved with high efficiency using several routes [116,117] (Figure 1 and Table 1): (1) renal artery, targeting the glomeruli and tubular epithelium. Podocytes within the glomerulus create slit diaphragms with diameters of only 10 nm. Results from a number of studies targeting the renal tubules via renal artery injection suggest that this glomerular filtration barrier can be temporarily disrupted by controlled hydrodynamic-pressure upon injection, potentially allowing the administration of large bioconjugates and nanoparticles [118]; (2) retrograde renal vein administration, predominantly targeting the renal tubules through the basolateral domain. Similar to what occurs in the renal artery, increased localized pressure in the renal capillaries creates transient pores on cell membranes, resulting in nucleic acid extravasation [18,22,61]; (3) retrograde ureteral administration, targeting the tubular epithelium; (4) intraparenchymal administration. Some reports have already demonstrated the suitability of using this route for the treatment of renal diseases by using gene therapy [116,119] and oligonucleotides delivered by viral vectors [30,32,33] (Table 1).

Although viral vectors are excluded by the glomerular filtration barrier, even those based on adeno-associated virus (AAV), which have the smallest capsid diameters (25 nm), the use of direct routes of administration has successfully achieved renal transduction [118,120,121]. This indicates the feasibility of using viral vectors and similar large-sized nanoparticles to deliver oligonucleotides. Similarly, local administration has also been evaluated for renal oligotherapeutics by direct delivery of naked siRNA via the renal artery in two different animal models, obtaining successful results [16,22] (Table 1). Other studies using direct renal administration of siRNAs are summarized in Yang et al. [60]. Interestingly, intraperitoneal administration of large drug bioconjugates and nanoparticles is also feasible for kidney targeting in certain circumstances. As shown by Yang et al. [20] in a murine model of UUO, chitosan NPs carrying a siRNA against cyclooxygenase type 2 (COX-2), were administered intraperitoneally and phagocytosed by the high number of macrophages present in the intraperitoneal cavity. Subsequently, macrophages that were recruited to the inflamed kidney showed reduced COX-2 immunoreactivity, finally translated in attenuated kidney damage and inflammation.

#### 3.2.2. Viral Delivery

An alternative to ON administration is the delivery of vectors containing expression cassettes coding for shRNA precursors, CRISPR Cas9, antagomirs, or miRNA mimics [119,122]. In that context, the use of viral vectors as delivery tools has shown several advantages compared to naked plasmid transfection, especially when pursuing long-term stable expression of the cassettes. The same strategies employed for their use in gene therapy can be adapted here, including the possibility of a rational design and modification of the viral particles to improve renal tropism [123], or kidney-specific promoters that increase specificity and expression in renal cells [124]. As previously mentioned, this strategy has already been successfully tested for gene therapy purposes [118,119].

Different types of viral vectors have been employed so far with the intention of delivering shRNA to the kidney by using different routes of administration (Table 1). Because of the low mitotic activity of kidney cells, lentiviral vectors are the most popular, as they are able to transduce post-mitotic cells. Some of the studies published included a lentiviral-based shRNA construct targeting split-and hairy-related protein-2 (SHARP-2) with a role in transplant rejection that significantly achieved gene silencing in rat mesangial cells after perfusion of isolated organs [29]. Zhou et al. [30] also demonstrated the feasibility of lentivirus-mediated shRNA targeting of collagen type I after renal parenchyma injection in rats. More recently, another similar paper achieved efficient and sustained gene silencing, maintained beyond 2 months, specifically in proximal and distal tubule cells after ultrasound-guided and repeated intraparenchymal injections of lentiviral vectors in vivo [32]. Some other studies using lentiviral vectors have been recapitulated in a recent review [119]. However, the use of lentiviral-based delivery systems is hampered due to safety concerns as result of insertional mutagenesis, the possibility to generate replication-competent recombinant lentivirus during vector production and their immunogenicity [61]. Similarly, the use of replication-deficient shRNA expression adenovirus vectors have also demonstrated promising results targeting the kidney, as shown by a recent study [33]. Nevertheless, comparable to what occurs with lentiviral vector development, their high immunogenicity severely limits their potential for clinical purposes [116]. Another alternative includes the use of AAV, whose absence of human pathogenicity make them attractive candidates. Indeed, a study carried out by Wang et al. [28] delivered an AAV carrying an shRNA targeting the mineralocorticoid receptor of the kidney in rats, achieving efficient silencing. Contrary to adenovirus or lentivirus, AAV present a smaller size and genetic-carrying capacity (25 nm/4,7 kb vs. 100 nm/36 kb and 130 nm/9kb respectively) [116]. However, this should not be an issue when packaging short oligonucleotide precursors. Although several studies have demonstrated the feasibility as of AAVs as vectors for efficient kidney delivery, it is important to note that their smaller size enabled leakage out of the kidney and transduction of off-target tissues [118,120]. Overall, the use of viral vectors for kidney delivery remains promising; however, further progress is needed to improve transduction and reduce off-target effects.

#### 3.2.3. Nanocarriers

The delivery potential of oligonucleotides can be enhanced by conjugation with nanocarriers of various types, which protect them from degradation, reduce kidney clearance by increasing drug size, target the drugs to specific cells or tissues and potentiate cell uptake and internalization. In general, these groups can be conjugated to any termini of the oligonucleotide, although the 5′ end is usually avoided in the case of siRNA since this region is important for the interaction with AGO2 [4,59]. Some of the moieties that have been described include: polyethylene glycol (PEG), which prolongs circulation half-life and bioavailability [90,125]; lipids, such as cholesterol, which promote interaction with lipoproteins in circulation [125]; peptides and proteins, such as antibodies, to enhance cell targeting and penetration [59,125,126]; sugars, such as N-acetylgalactosamine (GalNAc), which binds the asialoglycoprotein receptor (ASGR) with high affinity and facilitates liver-specific uptake [4,59] and oligonucleotides, such as aptamers [90,125]. Importantly, bioconjugates usually include acid-labile likers that allow fast disassembly and endosomal escape after cell entry. In some cases, small molecules with the ability to lyse the endosomal membrane, such as chloroquine or melittin, have been administered along with the drug or conjugated to it [115].

There are some studies that have already efficiently used such approaches in the pre-clinical assessment of oligotherapeutics against renal disease. Although not using covalent bioconjugates, Shimizu et al. [17] harnessed the electrostatic interactions between positively charged nanocarriers, polyethylene glycol-poly (l-lysine)-polymers, and negatively charged siRNA to facilitate delivery. After peritoneal systemic administration, the nanocarrier/siRNA efficiently targeted mesangial cells in the glomeruli and inhibited *Mapk1* mRNA in a murine model of glomerulonephritis. Another study intravenously administered an anti-miR107 complexed with atelocollagen in mice, which was efficiently transferred to renal peritubular endothelial cells, preventing tubular cell injury in an AKI model induced by sepsis [39]. Indeed, atelocollagen protein has been previously demonstrated to increase the permeability of endothelial cells [127]. In addition to the use of ON conjugation to nanocarriers to improve target delivery to the kidney, ON has also been used to alter pharmacokinetics, reducing renal accumulation and toxicity. A recent study by Wada et al. [128] used a cholesterol-GalNac dual conjugated ASO, which exhibited five times lower renal accumulation compared to the ASO modified only with GalNac, while liver gene silencing activity was maintained.

#### 3.2.4. Nanoparticles

The latest advances in nanotechnology and material science have emerged as an alternative solution to the challenge of oligonucleotide drug delivery. NPs offer many advantages, such as their high in vivo stability and retention due to decreased enzymatic degradation and sequestration by macrophages [129,130]. Importantly, their design can be tailored by adapting their physical and chemical characteristics to each intended purpose. The chemical composition of NPs usually includes immunochemical inert materials, which are nontoxic and biodegradable, such as cationic polymers that are able to condense high amounts of nucleic acids via electrostatic interactions. Another alternative is the use of lipid nanoparticles (LNPs) with enhanced cellular uptake and endosomal escape [4,59,131,132]. The use of liposomes for efficient kidney delivery of ONs was evaluated by Chae et al. in 2006 [53]. The authors combined the hemagglutinating virus of Japan (HVJ or Sendai virus) with liposome-based nonviral vectors to deliver a TFD in rats. HVJ-liposomes were also employed in a second study, showing comparable positive results for delivery of another TFD by renal artery administration in rats [54]. More recently, another group has also achieved co-delivery of two siRNAs against p38 mitogen-activated protein kinase (MAPK) and p65 by using glomerulus-targeting liposomal NP [26]. The use of NPs with specific biocompatible and nonimmunogenic formulations have been also assessed as kidney delivery platforms. In a recent study, the authors used ammonium-functionalized carbon nanotube (fCNT)–mediated transport of siRNA that selectively accumulated into renal proximal tubule cells in animal models of AKI after intravenous administration, which effectively silenced specific target genes [23]. These single-walled carbon nanotubes were particularly relevant because of their very favorable renal glomerular filtration and elimination profile, which allowed reabsorption by endocytosis in renal tubules.

Controlling the size and shape of the NPs can be critical for correct delivery. The size and shape of polymers comprises a range of assemblies, including linear and branched polymers, micelles, dendrimers or solid NPs, which can also present cubical, spherical, hexagonal, or rod-like shapes. LNPs generally present sizes of around 30–120 nm, wherein the nucleic acid can directly interact with lipids by electrostatic forces (lipoplexes) or can be encapsulated by a lipid bilayer (liposomes and exosomes) [4,132]. Indeed, controlling particle size could be crucial for improving cellular uptake, circulation half-life and kidney targeting. NPs of an average size of 100 nm have a longer half-life period than NPs of smaller sizes, which can be removed either by phagocytosis in the liver and spleen, or by renal excretion if they are smaller than 10 nm. This fact is extremely important to take into consideration when targeting the kidney. Indeed, research has shown that NPs with sizes of approximately 75 ± 25 nm targeted the renal mesangium, whereas larger NPs (>100 nm) could not pass the glomerular filter. Consequently, studies focusing on targeting renal tubule cells have designed NPs smaller than 10 nm, allowing them to pass the glomerular filtration barrier and be internalized by the epithelial cells [133]. Similarly, Han et al. [134] postulated that nanoparticles smaller than 250 nm tend to accumulate in the liver and spleen. Therefore, and in contrast to other studies utilizing small NPs for kidney delivery, the authors developed poly (lactic-co-glycolic acid) mesoscale nanoparticles (300–400 nm) that selectively localized up to seven times more efficiently in the kidney than in other organs, efficiently targeted renal proximal tubule cells and delivered an oligonucleotide against TLR9 that attenuated renal tubular fibrosis and inflammation in a model of ischemic AKI in mice. As the large size of mesoscale NPs would preclude glomerular filtration, the author suggest that these NPs are most likely transcytosed across the peritubular capillary endothelium. Additionally, Gao et al. [18] used low molecular weight chitosan/siRNA nanoparticles, which accumulated in renal proximal tubule cells in a process mediated by megalin-dependent endocytosis, demonstrating the importance of the size and molecular weight of the polymers for their specific accumulation in the kidneys. After kidney targeting by intravenous systemic administration in mice, siRNA achieved up to 50% silencing in aquaporin 1 expression.

Advances in nanotechnology have also allowed the development of DNA nanostructures as novel delivery platforms that can be readily loaded with nucleic acid drugs by base pairing and are highly biocompatible. These nanostructures, resembling nanoparticles, typically self-assemble by sequence complementarity and can be easily engineered with precise geometries and sizes as small as 20 nm. A very interesting property of DNA nanostructures is that they have been reported to not accumulate in the liver [4,130]. In fact, in vivo biodistribution of a small-sized DNA tetrahedron favored kidney-specific accumulation after intravenous administration in a mouse model [27]. The authors indicated that low opsonization with serum proteins reduced liver clearance and the small size of the nanostructure allowed it to pass though the glomerular filter and be endocytosed by tubular cells. Importantly, the authors have demonstrated the functionality of this platform by achieving intracellular delivery of p53 siRNA and improved markers of AKI in mice [27].

Chemical conjugation of the nanoparticles with different nanocarriers that increase circulation time and enhance cell uptake and specificity is another option. This includes the use of targeting ligands, such as antibodies or aptamers [4,59,131]. Indeed, several ligands such as E-selectin antibody, Ac2-26 peptide, cyclopeptide, or angiotensin I/II have been already employed to target their corresponding receptors in mesangial cells, podocytes or tubular cells [133]. Interestingly, the use of small molecules as ligands has also been evaluated. In a study carried out by Zuckerman et al. [21] polyatomic cyclodextrin nanoparticle uptake was enhanced in human and mouse mesangial cells in vitro by functionalizing them with mannose or transferrin. The authors were also able to effectively deliver a functional siRNA to the glomerular mesangium in mice.

#### 3.2.5. Aptamers

Although the availability of new technologies for targeted delivery of oligonucleotides has increased in the last decade, there is still scope for the development of new methods that allow us to target specific cell populations and tissues while reducing the widespread problem of off-targets. As explained in the previous chapter, aptamers can be considered as “chemical antibodies”, binding to their respective target molecules with high affinities and specificities, but presenting several advantages over them, such as easy manufacturing and modification, low immunogenicity and smaller size [86,90]. Thus, by taking advantage of their properties, cell or tissue-specific aptamers could be conjugated to siRNAs, miRNAs, ASOs, or other therapeutic aptamers which are specifically recognized and internalized by target cells, thereby improving the local concentration of the drug and its therapeutic efficacy [90,94,135]. However, despite the fact that this strategy has been widely employed in cancer and other diseases [135,136], there are no reports of this approach being used for kidney delivery of oligonucleotide drugs.

Alternative to the use of aptamers directly conjugated to ON-drugs, they can also be employed as nanocarriers bound to large nanoparticles. Currently, several types of nanoparticles have been successfully combined with cell-specific aptamers to deliver therapeutic agents to specific target tissues [95,135]. One example is the aptamer AS1411, which has been used as a nanocarrier for improved drug delivery for the specific targeting of cancer cells [137], including renal cancer [138,139]. Interestingly, aptamers have also been conjugated to viral vector capsid proteins in an attempt to improve viral tropism. This strategy includes conjugation reactions to amino acids, such as arginine or lysine, which are naturally present in the capsid proteins, or incorporating unnatural amino acids (UAA) into specific sites of the viral capsid proteins that allow later functionalization by simple click chemistry reactions [140,141]. The use of this approach has already shown promising results, improving transduction efficiency in a range of cell types in vitro [142,143,144]. Altogether, these approaches point to a new and promising direction for the development of oligotherapeutics with enhanced kidney tropism, although the development of novel renal specific aptamers and further in vivo characterization of these platforms is still required.

## 4. Conclusions and Perspectives

The use of oligonucleotide-based approaches for the treatment of renal disease is feasible and has been gaining more relevance over the recent years, as exemplified by the numerous studies shown in this review and two drugs (RG012 and RGLS4326) already undergoing clinical development. The studies that have been performed to date have demonstrated that oligonucleotide-based therapies are capable of reaching kidney cells in vivo, and, in some cases, conferring a therapeutic effect. However, the wide retention of the drugs by other organs and, consequently, the potential development of off-targets and side effects is still limiting their translation to the clinic. Moreover, even though drug biodistribution itself tends to favor kidney accumulation, it can act as a double-edged sword causing the development of kidney toxicities. Thus, although recent studies move forward in the correct direction, there is still scope for further development and characterization to improve the clinical translation from animal models to patients. The platforms reviewed here include a plethora of strategies, such as the use of small nanocarriers, nanoparticles, viral particles, and aptamers, which can be combined with more direct routes of administration that avoid unspecific drug retention, or the use of kidney-specific promoters to reduce off-targets. The use of these nanotechnological platforms has emerged as a promising tool for the future development of novel strategies that improve delivery efficiency and specificity, bringing us closer to the potential use of oligonucleotide-based therapies for renal diseases in the clinic.

## Figures and Tables

**Table 1:** Summary of preclinical studies targeting the kidney with oligonucleotide-based therapies.

STRATEGY	REFERENCE	RENAL TARGETS (Target; Cell/Tissue Type; Animal Model)	SEQUENCE (5′-3′)	CARRIER AND ROUTE OF ADMINISTRATION
siRNA	Molitoris et al., 2009 [15]	*Trp53*; PTECs; cisplatin-induced and ischemic AKI models in rats	GAAGAAAAUUUCCGCAAAA	Naked; IV
	Takabatake et al., 2009 [16]	*Egfp* and *Tgfb1*; glomeruli; glomerulonephritis model in rats	*Egfp*—GGCUACGUCCAGGAGCGCA *Tgfb1*—GUCAACUGUGGAGCAACACdTdT	Naked, RA
	Shimizu et al., 2010 [17]	*Mapk1;* glomeruli; glomerulonephritis model in mice and rats	UGCUGACUCCAAAGCUCUGdTdT	Polyion complex nanocarriers; IP
	Gao et al., 2014 [18]	*Aqp1*; PTECs; mice	CGCAACUUCUCAAACCACUTT	Chitosan NPs; IV
	Morishita et al., 2014 [19]	*Smad4*; tubulointerstitium and tubule epithelial cells; renal fibrosis model in mice	GAUGAAUUGGAUUCUUUAATT	Naked; IV
	Yang et al., 2015 [20]	*Cox2*; peritoneal macrophages recruited to the kidney; UUO model in mice	GGAUUUGACCAGUAUAAGUTT	Chitosan NPs; IP
	Zuckerman et al., 2015 [21]	*Egfp;* glomerular mesangium; mice	GGCUACGUCCAGGAGCGCACC	Polycationic cyclodextrin NPs functionalized with mannose and transferrin; IV
	Zheng et al., 2016 [22]	*Fas*, *C3* and *RelB*; glomeruli and medullar tubule cells; ischemic AKI in mice	*Fas*—GUGCAAGUGCAA ACCAGAC *C3*—GUGCAAGACUUCCUAAAGA *RelB*—GGAAUCGAGAGCAAACGAA	Naked; RA
	Alidori et al., 2016 [23]	*Ctr1, Trp53* and *Mep1b*; cortex and PTECs; AKI model in mice	*Ctr1*—GGCAUGAACAUGUGAAUUGCUGGTT *Trp53*—AGGAGUCACAGUCGGAUAUCAGCCT *Mep1b*—GGAAUUGACCAAGACAUAUUU GATA	Fibrillar carbon nanotubes (fCNT); IV
	Eadon et al., 2017 [24]	*Lrp2*; PTECs, mice		Naked or lipid-base transfection; IV
	Narváez et al., 2019 [25]	*Cd40*; tubulointerstitium; UUO model in mice	GUGUGUUACGUGCAGUGACUU	Naked; IV
	Wang et al., 2020 [26]	*p38α* MapK and *p65*; glomerular mesangium and peritubular endothelial cells; glomerulonephritis model in mice	*p38α*—GGUCACUGGAGGAAUUC *p65*—GCGACAAGGUGCAGAAAGA	Liposomal NPs, IV
	Thai et al., 2020 [27]	*Trp53*; tubular epithelial cells; AKI model in mice	GAGAAUAUUUCACCCUUCA	DNA nanostructure; IV
shRNA plasmid	Wang et al., 2006 [28]	*Mr;* cortical tubule cells; renal hypertension and damage model in rats	CCAACAAGGAAGCCTGAGC	AAV; IV
	Shou et al., 2009 [29]	*Sharp2*; T-cells; transplantation model in rats	ACCCGAACATCTCAAACTTA	Lentivirus; ex vivo perfusion
	Zhou et al., 2011 [30]	*ColI*; cortex; rats	GCAACCTGGATGCCATCAA	Lentivirus; RP
	Fujino et al., 2013 [31]	*Trp53*: cortex and medullar tubule cells; ischemic AKI model in mice		Cationic polymer; RA
	Espana-Agusti et al., 2015 [32]	*Tsc1* and *Luc*; PTECs, DTECs and interstitium; mice	*Tsc1*—CGGAAGAAGCTGCAATATCTAA *Luc*—CCGCCTGAAGTCTCTGATTAA	Lentivirus; RP
	Xu et al., 2020 [33]	*Yap* and *Klf4*; renal tubules; ischemic AKI model in mice		Adenovirus; RP
saRNA	Zeng et al., 2018 [34]	*Trpv5*; medullar tubule cells; calcium crystal formation model in rats	AAGGGTCTCATGATTTCTCTA	Naked; RU
miRNA antagomir	Chau et al., 2012 [35]	miR21; PTECs; UUO model in mice		Naked; IP
	Putta et al., 2012 [36]	miR192; cortex and glomeruli; DN model in mice	GGCTGTCAATTCATAGGTCAG	Naked; SC
	Li et al., 2014 [37]	miR204; cortex and medulla; candidemia-induced AKI model in mice	AGGCAUAGGAUGACAAAGGGAA	Naked; IV
	Gomez et al., 2015 [38]	miR21; PTECs, Alport nephropathy mouse model		Naked, SC
	Wang et al., 2017 [39]	miR107; peritubular endothelial cells; septic AKI model in mice		Complexed with atelocollagen; IV
	Wilflingseder et al., 2017 [40]	miR182-5p; cortex and medulla; ischemic AKI model in mice, rats and pigs		Naked; IV (mice and rats), ex vivo perfusion (pig)
	Wei et al., 2016 [41]	miR489; tubular epithelial cells; ischemic AKI model in mice		Naked; IV
	Wei et al., 2018 [42]	miR668; tubular epithelial cells; ischemic AKI model in mice		Naked; IV
	Lee et al., 2019 [43]	miR17; PTECs; ADPKD mouse model	GUUUCACGA	Naked; SC
	Luan et al., 2020 [44]	miR150; cortex and medulla; renal fibrosis model in mice	UACAAGGGUUGGGAG	Naked; IV
miRNA mimic	Li et al., 2014 [37]	miR204 and miR211; cortex and medulla; candidemia-induced AKI in mice	miR204—UCCCGGUAAUCCCUUACCUGGUU CCCUUCCUU miR211—UCCCGGCUUUCCCUUACCUGGUUUUCCCCCUU	Naked, IV
	Wei et al., 2018 [42]	miR668; cortex and medulla; ischemic AKI model in mice		Lipid-based transfection reagent; IV
	Zhu et al., 2019 [45]	miR199a-3p; tubular epithelial cells; ischemic model AKI in mice		Exosomes; IV
ASO	Shi and Siemann [46]	*Vegf*; Caki-I RCC cell line; xenograft model in mice	CTCACCCGTCCATGAGCCCG	Naked; IV
	Daniel et al., 2003 [47]	*Tsp1*; glomeruli; glomerulonephritis model in mice	*Tsp1-1*—TTCTCCGTTGTGATTGAA *Tsp1-2*—CACCTCCAATGAGTT	Naked by electroporation or HVJ-liposomes; RA
	Kausch et al., 2004 [48]	*Ki67*; Renca cells; RCC orthotopic model in mice	ACCAGGTGAGCCGAGGACGCCAT	Naked, IP
	Guha et al., 2007 [49]	*Ctgf*; PTECs and mesangial cells; DN model in mice	CCACAAGCTGTCCAGTCTAA	Naked; SC
	Wang et al., 2012 [50]	*Kras*; tubular epithelial cells; UUO model in rats	*Kras-1*—ATTCACATGACTATACACCT *Kras-2*—CACACTTATTCCCTACTAGG	Naked; SC
	Ravichandran et al., 2014 [51]	*mTORC*; tubular epithelial cells; PKD mouse model	TCCACTTTTCACAGCACTGC	Naked, IP
	Ravichandran et al., 2015 [52]	*Agt*; tubular epithelial cells; PKD mouse model	TCTTCCACCCTGTCACAGCC	Naked, IP
TFD	Chae et al., 2006 [53]	*Sp1*; tubulointerstitium; UUO model in rats	GGGGCGGGGC	HVJ-liposomes; RV
	Tomita et al., 2007 [54]	*E2f*; glomeruli; rats		HVJ-liposomes; RA
Aptamers	Matsui et al., 2017 [55]	RAGE; kidney, heart, eyes, testis; DN model in rats	CCTGATATGGTGTCACCGCCGCCTTAGTATTGGTGTCTAC	Naked; IP
	Um et al., 2017 [56]	Periostin; medulla; DN model in mice		PEG-conjugated; IP
	Taguchi et al., 2018 [57]	RAGE; glomeruli; hypertensive mouse model	CATTCTTAGATTTTTGTCTCACTTAGGTGTAGATGGTGAT	Naked; SC
	Zhang et al., 2018 [58]	RCC 786-O cells; xenograft model in mice	ACTCATAGGGTTAGGGGCTGCTGGCCAGATATTCAGATGGTAGGGTTACTATGA	Naked; IV

Abbreviations: proximal tubule epithelial cells (PTECs); distal tubule epithelial cells (DTECs); diabetic nephropathy (DN); unilateral ureteral obstruction (UUO); acute kidney injury (AKI); renal cell carcinoma (RCC); intravenous administration (IV); subcutaneous administration (SC); intraperitoneal administration (IP); Renal artery administration (RA); retrograde renal vein administration (RV); renal parenchyma administration (RP); retrograde ureteral administration (RU); nanoparticles (NPs); adeno-associated virus (AAV); hemagglutinating virus of Japan (HVJ); antisense oligonucleotide (ASO); transcription factor decoy (TFD); sequences that are not listed within the table were not specified or could not be found within the corresponding article, or are under the protection of a patent.
